# Supplementary figures and images for: The Defined TLR3 Agonist, Nexavant, Exhibits Anti-Cancer Efficacy and Potentiates Anti-PD-1 Antibody Therapy by Enhancing Immune Cell Infiltration
Source: Cancers (Basel). 2023 Dec 8;15(24):5752. doi: 10.3390/cancers15245752 (PMC10741573; doi:10.3390/cancers15245752)

Figure 1B

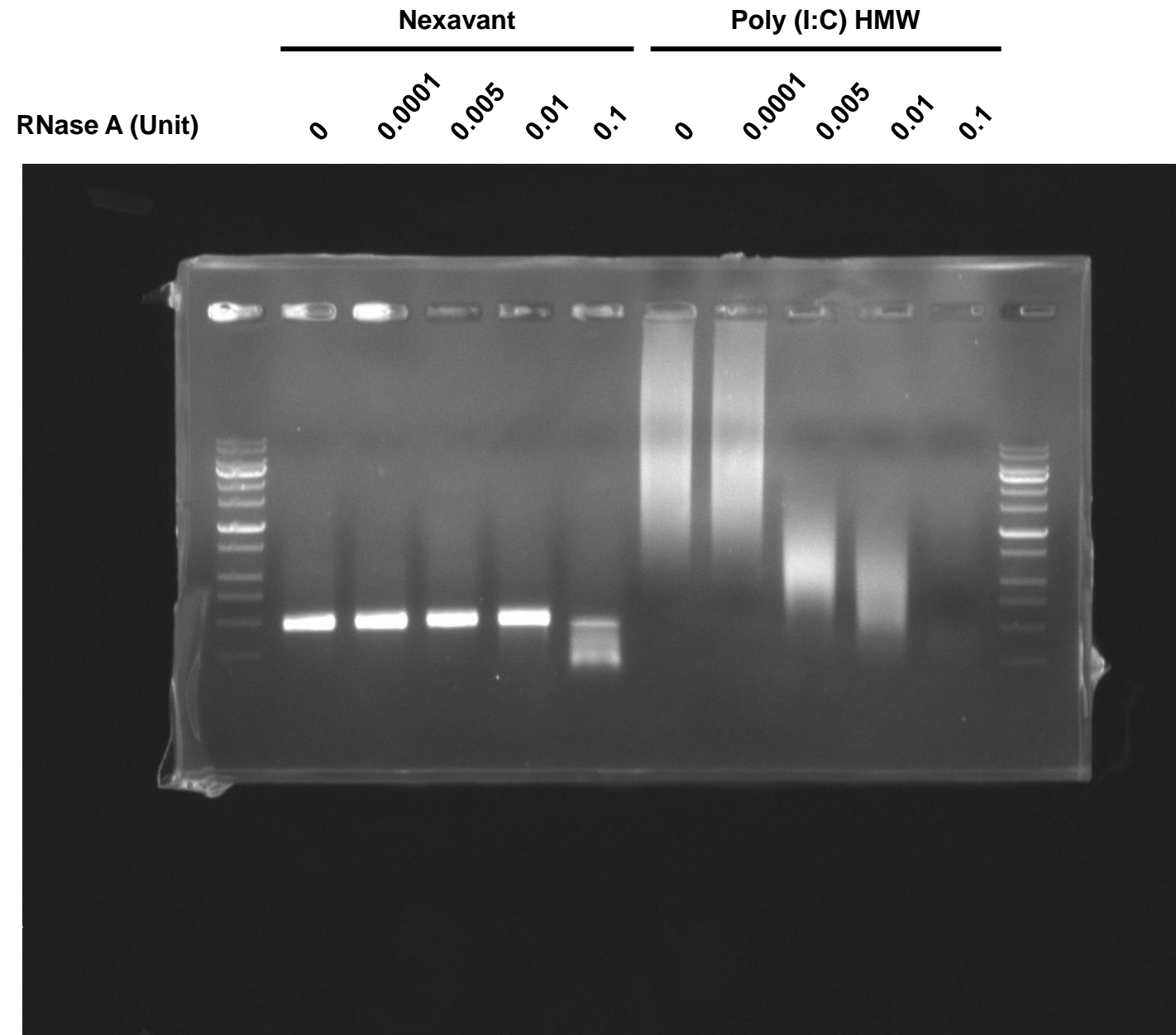

Figure 1C

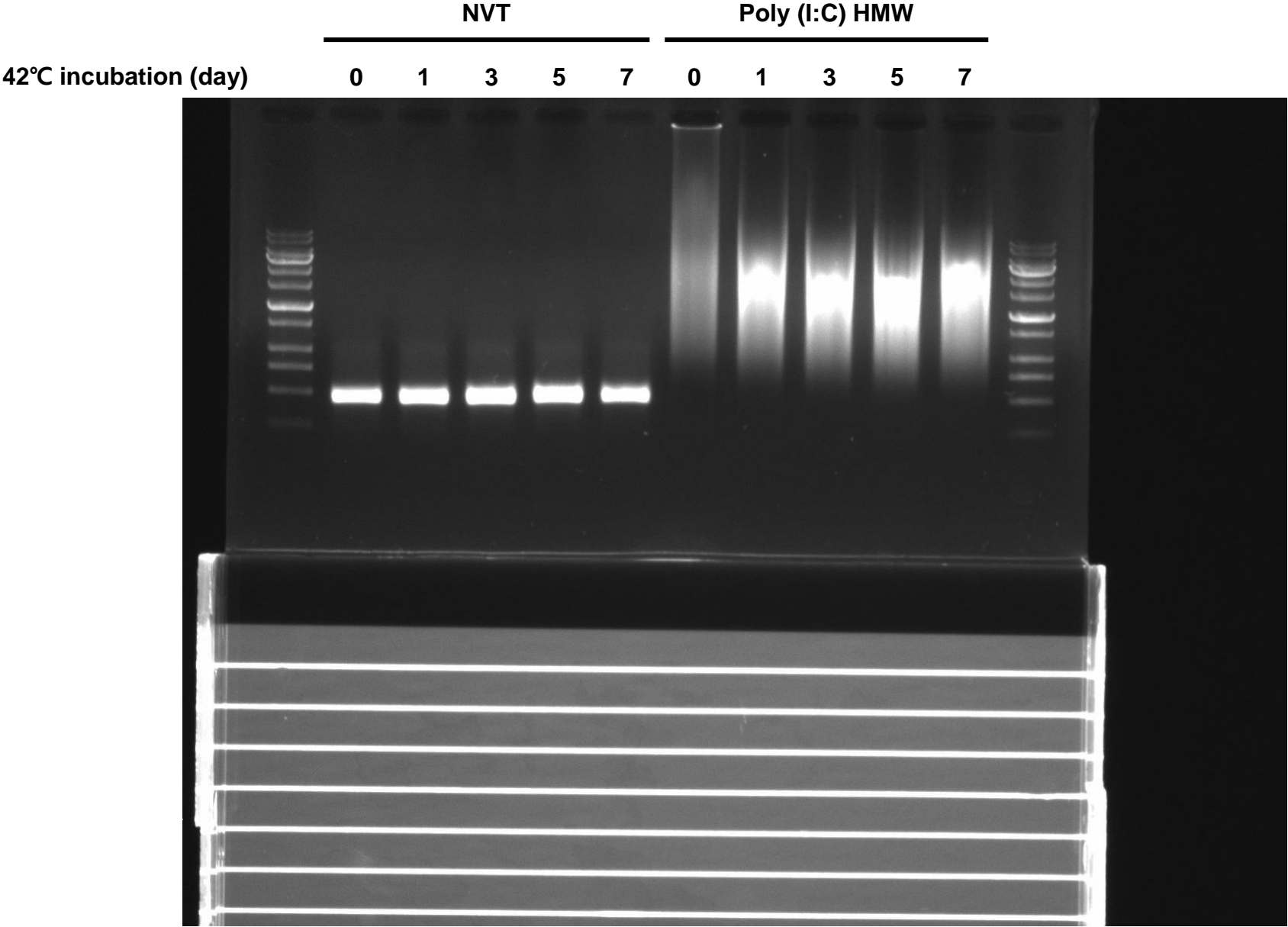

Figure 7B

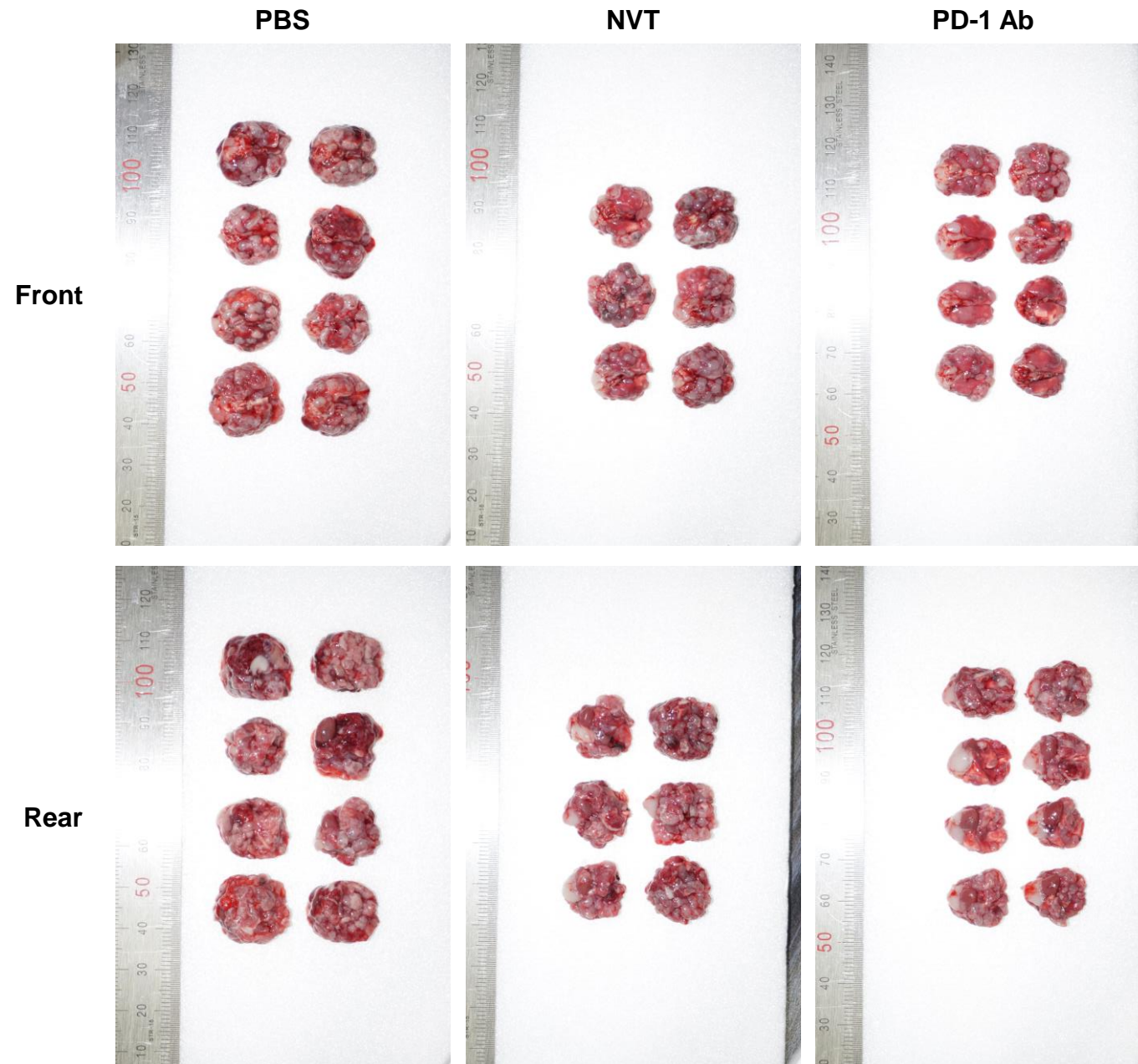

Figure 7F

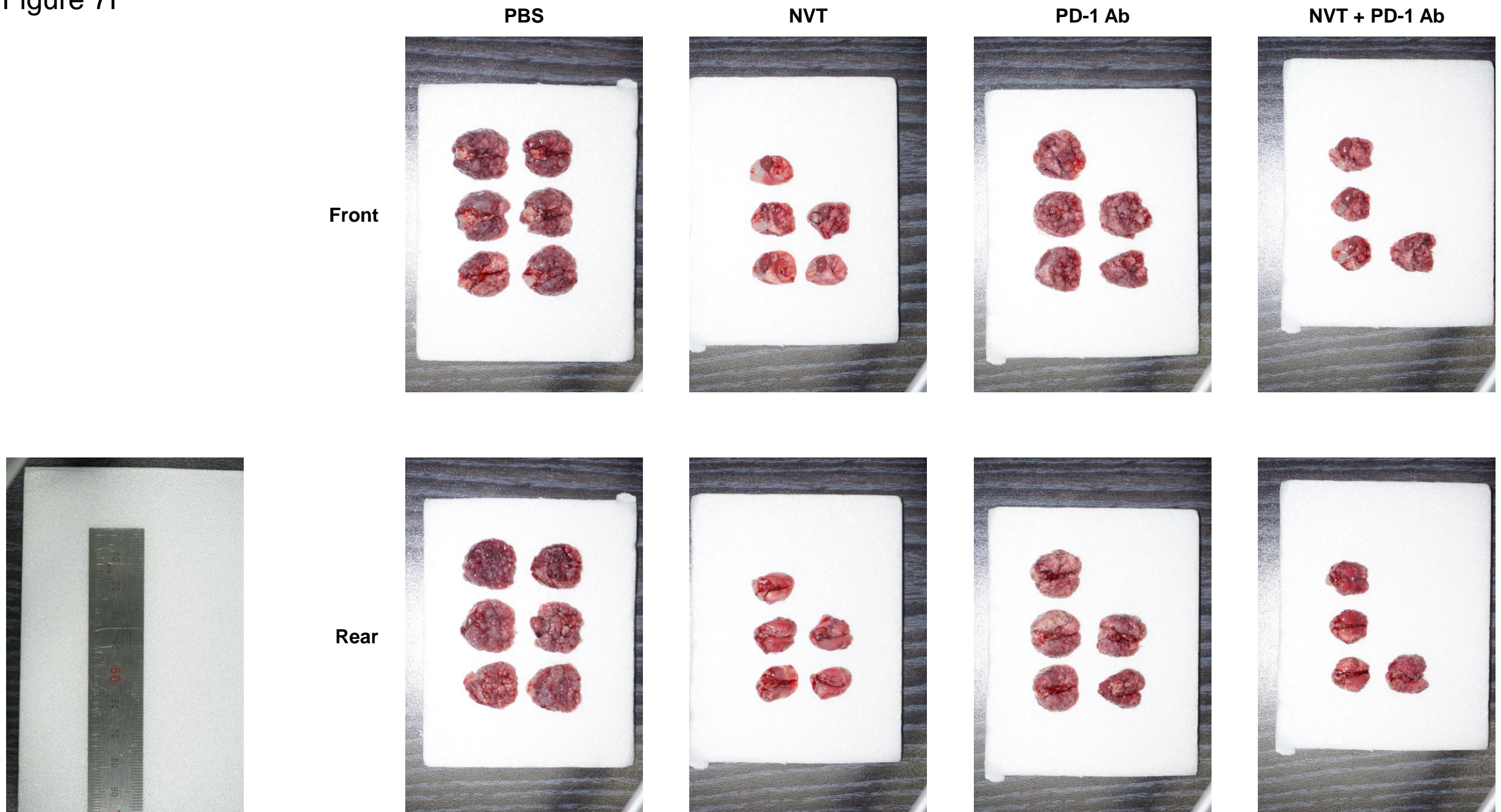

Supplement: Supplementary file 1 [file cancers-15-05752-s001.zip › cancers-2710442-supplementary.pdf]
